# Citrulline a More Suitable Substrate than Arginine to Restore NO Production and the Microcirculation during Endotoxemia

**DOI:** 10.1371/journal.pone.0037439

**Published:** 2012-05-29

**Authors:** Karolina A. P. Wijnands, Hans Vink, Jacob J. Briedé, Ernst E. van Faassen, Wouter H. Lamers, Wim A. Buurman, Martijn Poeze

**Affiliations:** 1 Department of Surgery, Maastricht University Medical Center, Maastricht, The Netherlands; 2 NUTRIM School for Nutrition, Toxicology and Metabolism, Maastricht, The Netherlands; 3 Department of Physiology, Maastricht University Medical Center, Maastricht, The Netherlands; 4 CARIM Cardiovascular Research Institute of Maastricht, Maastricht, The Netherlands; 5 Department of Toxicogenomics, Maastricht University Medical Center, Maastricht, The Netherlands; 6 GROW School for Oncology and Developmental Biology, Maastricht, The Netherlands; 7 Department of Nephrology, Leiden University Medical Center, Leiden, The Netherlands; 8 Department of Anatomy and Embryology, Maastricht University Medical Center, Maastricht, The Netherlands; National Institute of Agronomic Research, France

## Abstract

**Background:**

Impaired microcirculation during endotoxemia correlates with a disturbed arginine-nitric oxide (NO) metabolism and is associated with deteriorating organ function. Improving the organ perfusion in endotoxemia, as often seen in patients with severe infection or systemic inflammatory response syndrome (SIRS) is, therefore, an important therapeutic target. We hypothesized that supplementation of the arginine precursor citrulline rather than arginine would specifically increase eNOS-induced intracellular NO production and thereby improve the microcirculation during endotoxemia.

**Methodology/Principal Findings:**

To study the effects of L-Citrulline and L-Arginine supplementation on jejunal microcirculation, intracellular arginine availability and NO production in a non-lethal prolonged endotoxemia model in mice. C57/Bl6 mice received an 18 hrs intravenous infusion of endotoxin (LPS, 0.4 µg•g bodyweight^−1^•h^−1^), combined with either L-Citrulline (6.25 mg•h-1), L-Arginine (6.25 mg•h^−1^), or L-Alanine (isonitrogenous control; 12.5 mg•h^−1^) during the last 6 hrs. The control group received an 18 hrs sterile saline infusion combined with L-Alanine or L-Citrulline during the last 6 hrs. The microcirculation was evaluated at the end of the infusion period using sidestream dark-field imaging of jejunal villi. Plasma and jejunal tissue amino-acid concentrations were measured by HPLC, NO tissue concentrations by electron-spin resonance spectroscopy and NOS protein concentrations using Western blot.

**Conclusion/Significance:**

L-Citrulline supplementation during endotoxemia positively influenced the intestinal microvascular perfusion compared to L-Arginine-supplemented and control endotoxemic mice. L-Citrulline supplementation increased plasma and tissue concentrations of arginine and citrulline, and restored intracellular NO production in the intestine. L-Arginine supplementation did not increase the intracellular arginine availability. Jejunal tissues in the L-Citrulline-supplemented group showed, compared to the endotoxemic and L-Arginine-supplemented endotoxemic group, an increase in degree of phosphorylation of eNOS (Ser 1177) and a decrease in iNOS protein level. In conclusion, L-Citrulline supplementation during endotoxemia and not L-Arginine reduced intestinal microcirculatory dysfunction and increased intracellular NO production, likely via increased intracellular citrulline and arginine availability.

## Introduction

XEndotoxemia and inflammatory conditions, such as sepsis are associated with multiple-organ failure. This organ failure is caused by a dysfunctional organ perfusion at the microcirculatory level [1]–[3], which makes organ perfusion an important therapeutic target [4], [5]. Endotoxemia and inflammation-induced perturbations in organ perfusion are characterized by redistribution of blood flow between and within organs at the microcirculatory level and lead to ischemia in critical tissues such as lung, liver and gut [6]–[8]. Especially the villi of the small intestine are sensitive to a decreased blood flow and suffer from microcirculatory disturbances during endotoxemic conditions such as severe inflammation and sepsis [9]–[11]. Previous experimental and clinical studies demonstrated that hepatosplanchnic hypoperfusion is related to intestinal mucosal damage, impaired tissue oxygenation and an increase in inflammatory mediators [6], [12]–[14].

**Figure 1:**
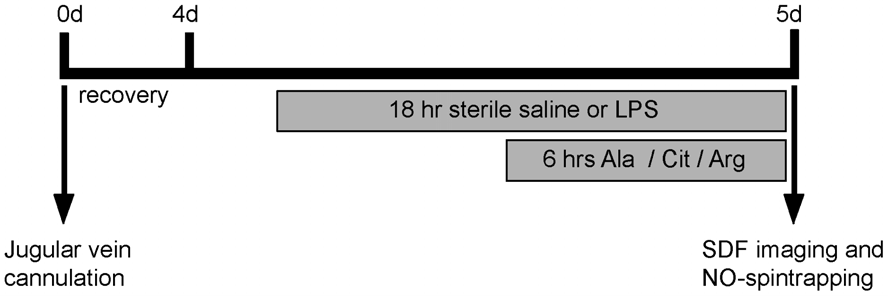
Experimental set up of the prolonged endotoxemia model. Mice were fitted with a jugular vein cannula at t = 0 d. After 4 days (t = 4 d) an 18hours continuous infusion with lipopolysaccharides (LPS) was started, which was combined during the last 6 hours with an infusion of Citrulline (Cit), Arginine (Arg) or an isonitrogenous quantity of the placebo Alanine (Ala). After completing the treatment, sidestream dark-field (SDF) imaging was used to quantify the microcirculation in the jejunal villi or Nitric Oxide (NO) spin trapping with iron-diethyldithiocarbamate (DETC) complexes to measure NO production *in vivo*.

An imbalance in Nitric Oxide (NO) metabolism at the cellular level, especially the endothelium, is thought to play a crucial role in the development of endotoxemic-related microcirculatory disturbances, such as in sepsis [15]–[18]. NO is produced by the conversion of arginine into citrulline by one of the three isoforms of nitric-oxide synthase (NOS) and is thought to depend largely on extracellular arginine availability, which is decreased during endotoxemia and sepsis [15], [17], [19]. Several mechanisms were proposed to explain arginine deficiency in diseases with an increased arginine catabolism such as endotoxemia and sepsis. Especially, enhanced activity of Arginase-1 [20], [21] and iNOS (NOS2), but also diminished *de novo* arginine synthesis from citrulline and a diminished *de novo* citrulline synthesis are relevant mechanism which contribute to arginine deficiency [17], [19], [22]. iNOS can produce excessive amounts of NO during endotoxemia, which contributes to the decreased arterial pressure and abnormal distribution of the blood flow in the microcirculation [18], [23], [24]. The resulting low plasma arginine levels may lead to decreased intracellular substrate availability in the microvasculature for eNOS (NOS3) [17]. This decreased substrate availability in combination with endotoxemia-induced eNOS downregulation results in decreased eNOS-mediated NO production, which is considered to result in the microcirculatory disturbances in sepsis [25]. Interestingly, L-Arginine supplementation in prolonged endotoxemia and sepsis failed to increase the intracellular NO production despite increased arginine plasma availability [26]–[30], hypothetically because the intracellular arginine does not increase during L-Arginine supplementation [15], [22].

The co-localization of eNOS and ASS in the caveola of the endothelial cells indicates a tight intracellular coupling between citrulline, arginine and the NO metabolism [27]. The presence of ASS in endothelial cells offers the opportunity to use L-Citrulline supplementation to increase intracellular endothelial arginine availability for NO production [16], [22], [27] and, thereby, to ameliorate microcirculatory dysfunction. We therefore hypothesize that L-Citrulline supplementation could improve splanchnic microcirculation by increasing the intracellular citrulline, arginine and NO availability. Therefore, we investigated the effects of L-Citrulline and L-Arginine supplementation on the microcirculation, the intracellular arginine availability and NO production in a murine prolonged endotoxemia model.

## Materials and Methods

Additional details about animals and procedures are provided in the **Methods S1.**

### Animals

Sixty-five male C57/Bl6 mice with a mean weight of 27 g (range 26–28.5) were obtained from the Central Animal Facilities of the Maastricht University Medical Center. The protocol was approved by the Committee on the Ethics of Animal Experiments of the Maastricht University Medical Center (Permit Number: 2008-042 and 2010-172). (**additional details in the online Methods S1**).

**Figure 2:**
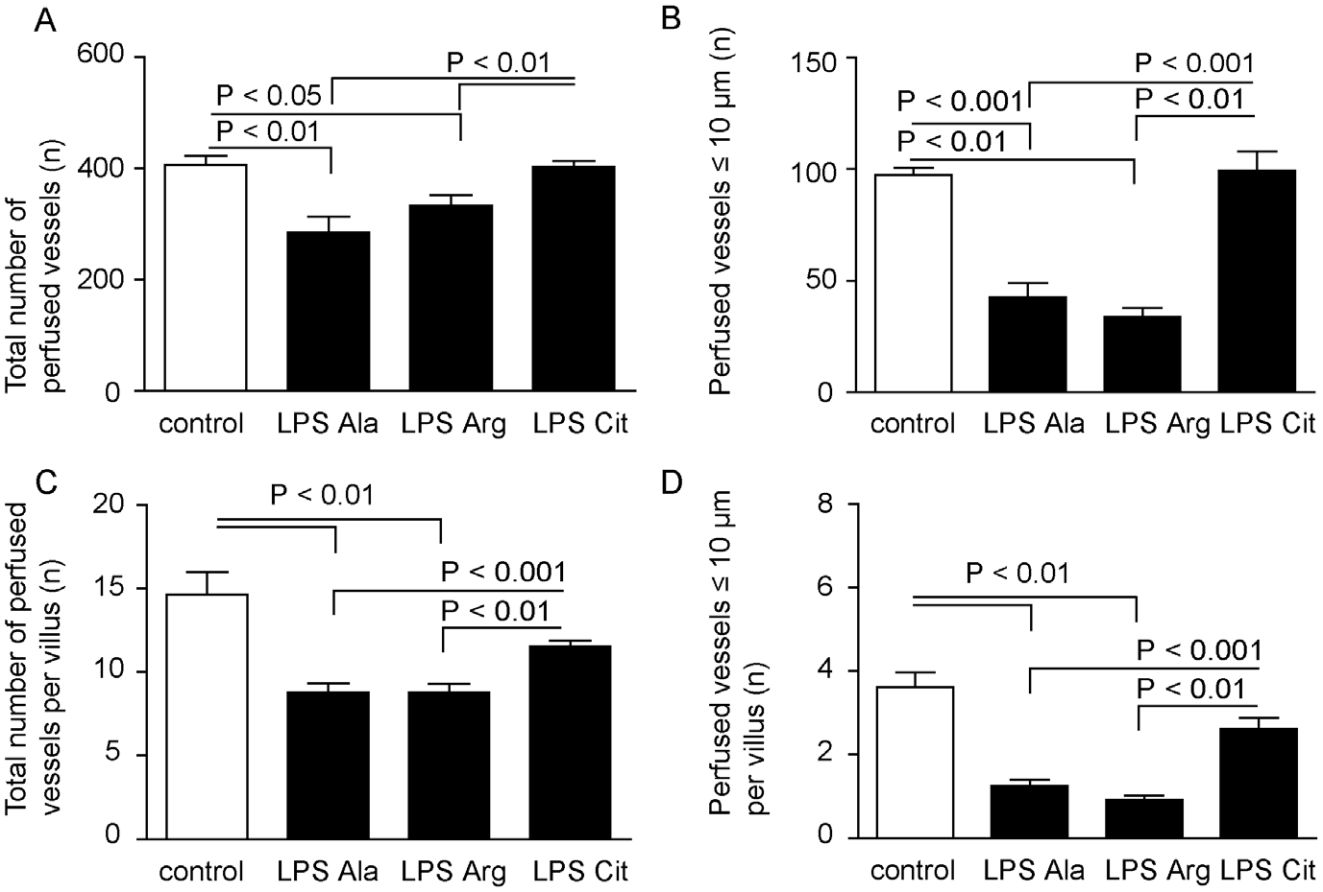
Circulation measurements with SDF imaging in the control, LPS-Ala, LPS-Arg and LPS-Cit groups. (A) L-Citrulline supplementation improved the number (mean±SEM) of measurable perfused vessels in the jejunal microcirculation during endotoxemia, (B) in particular of vessels with a diameter ≤10 µm (P<0.01), compared to LPS-Ala and LPS-Arg supplemented animals. (C) The total number of perfused vessels per villus and (D) microcirculatory vessels with a diameter ≤10 µm per villus were also significantly lower in the LPS-Ala and LPS-Arg supplemented animals compared to the control and LPS-Cit group.

### Experimental Protocol

Four days prior to the experimental period, mice received a jugular vein catheter (Silclear tubing, MEDNET, Münster, Germany) which was fixed to the head with glass ionomer cement (FuijCEM Automix, Instech Solomon, Plymouth Meeting, PA). Mice (n = 65) were randomly allocated to an 18 hour infusion with either sterile 0.9% saline (n = 26) or lipopolysaccharide (LPS; E. Coli O55:B5, Sigma Aldrich, St. Louis, MO) (n = 39) (**Figure 1**). LPS (0.4 µg•g bodyweight^−1^•h^−1^) was administered with a continuous flow rate of 83 µL/h. In the final 6 hours of the LPS infusion, L-Citrulline (1.25 g/kg total; n = 13; “LPS-Cit” group), L-Arginine (1.25 g/kg total; n = 13; “LPS-Arg” group) or an isonitrogenous dosage of the placebo amino acid L-Alanine (2.5 g/kg total; n = 13; “LPS-Ala” group) was added to the LPS infusate. All mice survived the experiments. To determine the clinical manifestations of endotoxemia a semi-quantitative evaluation score was developed. Contents of the score were based on the specific endpoints used in previous endotoxemia models in small laboratory animals of our group [31], [32] and from the human endpoints for infectious animal models [33]. These characteristic clinical manifestations of endotoxemia (piloerection/erection of the fur, exudates around the eyes and nostrils, lethargy, hypothermia, diarrhea and diminished locomotor activity) were scored by the animal care taker, blinded for treatment. A 2-points (absent/present) score was used for lethargy, hypothermia and diarrhea, and a 3-points score (absent/moderate/severe) for the evaluation of piloerection/erection of the fur, exudates around the eyes/nostrils and diminished locomotor activity.

The L-Citrulline and L-Arginine dosages were extrapolated from the arginine dosages used in both porcine endotoxemia [34] and a human arginine supplementation study (Luiking, Poeze, Deutz, unpublished data), taking in consideration that the nitrogen requirements in rodents are ten times higher than in humans [35], [36].

The control group was treated with 0.9% saline and L-Alanine (n = 13; “control” group). To investigate the role of L-Citrulline supplementation during physiological conditions mice were treated with 0.9% saline and L-Citrulline (n = 13, “NaCl-Cit”-group, data displayed in the online supplemental material). In total, mice received 1.5 mL fluid intravenously during the experimental period. Throughout final experiments (microcirculation, NO measurements, blood/tissue sampling), body temperature was maintained at 37°C using a heating pad and an infrared heating lamp with temperature controller connected to a rectal probe (Technical Service, Maastricht University). The mean arterial pressure (MAP) was measured during the microcirculatory measurements. In brief, a polyethylene tubing was inserted in the right carotid artery and connected to a low-volume pressure transducer to register the MAP (**additional details in the online Methods S1**).

### Microcirculation Measurements

After the 18 hours infusion, mice (n = 40) were anaesthetized and a small segment of the jejunum was exteriorized after a midline incision in the abdomen. A longitudinal incision in the jejunal segment was made to microscopically visualize the microcirculation in the mucosa with a sidestream dark-field (SDF) imager [37], [38]. The surgical procedure is based on the technique used by Massberg et al. [39] who used intravital fluorescence microscopy to evaluate the mucosal jejunal microcirculation. A specially designed stand was used to stabilize the SDF imager and to prevent pressure on the tissue or camera movement during the measurements. In addition, the microcirculation, defined as number of perfused microcirculatory vessels (diameter ≤10 µm) and the total number of perfused vessels (1 µm≤diameter≤50 µm) were measured and analyzed with Image J and Matlab. Furthermore, the characteristics of the microcirculation according to de Backer et al [2], [40], [41] were analyzed using the Automated Vascular Analysis software 3.0 (Microscan, Amsterdam, The Netherlands). Since Ava 3.0 was developed to analyze the sublingual microcirculation, adjustments as suggested by de Backer et al were made to analyze the jejunal microcirculation [40]. In brief, the average microvascular flow index (MFI), the determination of the predominant type of flow in the villi in the four quadrants of the image was determined (described as 0 = absent, 1 = intermittent, with at least 50% of the time no flow, 2 = sludging, 3 = normal or 4 = hyperdynamic flow) [2]. The percentage of perfused villi (%) was calculated based on the number of perfused villi per field divided by the total number of villi per field. The total vessel density (TVD; mm/mm^2^) was calculated as the number of vessels crossing the arbitrary lines in Ava 3.0 divided by the total length of the lines. The perfused vessel density (PVD; mm/mm^2^) and proportion of perfused vessels (PPV; %) were calculated based on the TVD and perfusion of these vessels [40], [41] (**additional details in the online Methods S1**).

**Figure 3:**
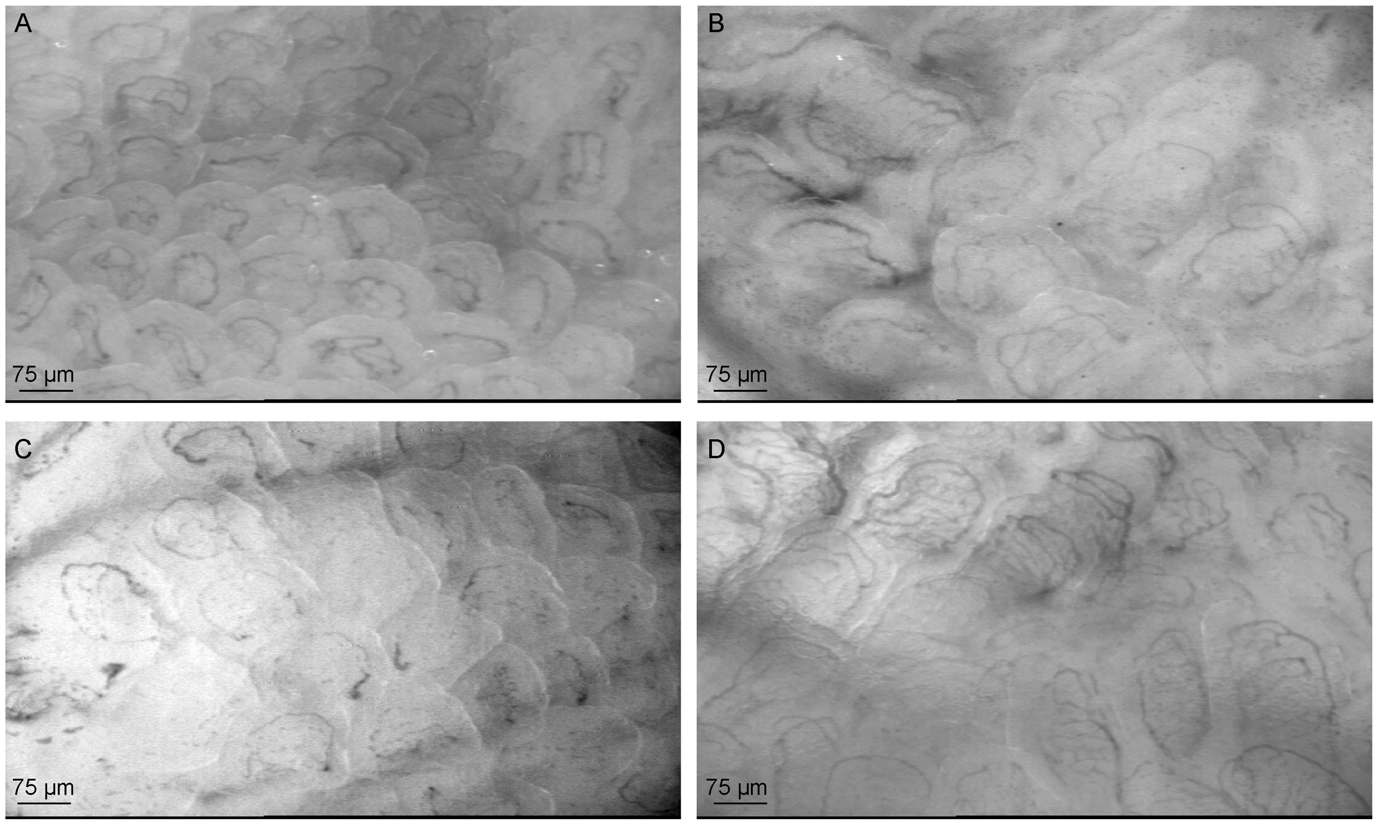
Representative live images of the microcirculatory measurements in jejunal villi with SDF imaging. (A) Representative live image of the jejunal microcirculation in a control mouse. (B) Representative live image of the jejunal microcirculation in a LPS-Ala treated mouse which shows only perfusion of the larger vessels. (C) Representative live image of a LPS-Arg treated mouse which shows a comparable perfusion pattern as the LPS-Ala treated mouse. (D) Representative live image of a LPS-Cit treated mouse, which’s shows more small perfused vessels per villus.

### Blood Sampling, Sample Processing and Measurements of Amino Acids

After the microcirculatory measurements, mice were sacrificed through a cardiac puncture to collect 600–800 µL arterial blood for amino acid determination. After sampling, blood was collected in pre-chilled, heparinized microvettes® (Sarstedt, Nϋmbrecht, Germany) on ice and centrifuged immediately (4°C for 15 minutes at 8,500 g) to obtain plasma. For determination of plasma amino-acid concentrations, 100 µL plasma was added to 200 µL acetonitrile, for deproteinization, vortexed and stored at -80°C until further analysis. To determine tissue amino-acid concentrations, frozen homogenized tissue samples were added to 250 µL of 5% sulfosalicylic acid and 0.1 g glass beads (1.0 mm diameter) for deproteinization, beaten for 30 seconds at maximum speed with the mini-beadbeater (Biospec products) and stored at −80°C until further analysis. Plasma and tissue arginine, citrulline and ornithine concentrations were measured with a high-performance liquid chromatography (HPLC) after automated pre-column derivatization with o-phthaldialdehyde as previously described [42].

### In vivo Tissue NO Measurements

In brief, the *in vivo* NO levels in jejunal tissues (n = 30) were quantified by NO-trapping with Fe^2+^-dithiocarbamate complexes as mono-nitrosyl iron complexes (MNIC) [43]. NO concentrations were calculated from the height of the three-line NO amplitude with Bruker WINEPR software as described [43] (**additional details in the online Methods S1**).

### Protein Isolation and Western Blot Analysis

Protein in the tissue samples was isolated with the AllPrep DNA/RNA/Protein kit (Qiagen, Hilden, Germany) according to the manufacturer’s protocol. Western blot membranes were incubated overnight with rabbit polyclonal anti-mouse iNOS (Abcam, Cambridge, MA) or rabbit polyclonal anti-mouse phosphorylated eNOS (Ser1177) (Cell signaling technology, Danvers, MA) at 4**°**C and incubated with the proper secondary antibody at room temperature for 1 hour, before capturing the signals on X-ray film using a chemiluminescent reaction (**additional details in the online Methods S1**).

### Statistical Analysis

Statistical analysis of the data was performed using SPSS 15.0 (SPSS, Chicago, IL). In case of Gaussian distribution one-way ANOVA with post-hoc Bonferroni correction between groups was conducted. A non-parametric Kruskal-Wallis test was performed to determine significance in case of a non-Gaussian distribution. A two-sided P–value<0.05 was considered as statistical significant. Data are represented as mean and standard error of the mean (SEM).

## Results

### Model Characteristics

The characteristic clinical manifestations of endotoxemia (piloerection/erection of the fur, exudates around the eyes and nostrils, hypothermia, lethargy, diarrhea and diminished locomotor activity) were present in all three LPS-treated groups. However, these signs were more pronounced in the LPS-Ala and LPS Arg group than the LPS-Cit group (See **Figure S1**). The average core temperature of the mice prior to the start of the microcirculatory measurements was significantly lower in the LPS-Ala (33.8±1.7°C) and the LPS-Arg groups (33.8±1.0°C) than in the control group (35.8±0.7°C; both P<0.05) and the LPS-Cit group (35.9±1.0°C; both P<0.05). Prior to the microcirculatory measurements an infrared heating lamp with temperature controller was used to increase the core temperature of the mice, which resulted in stabilization of the core temperature during the microcirculatory measurements. The average core temperature of the mice during the microcirculation measurements did not differ between groups (36.9±0.1°C in the control, 35.0±2.1°C in the LPS-Ala group, 35.7±0.5°C in the LPS-Arg and 36.2±0.9°C in the LPS-Cit group).

**Figure 4:**
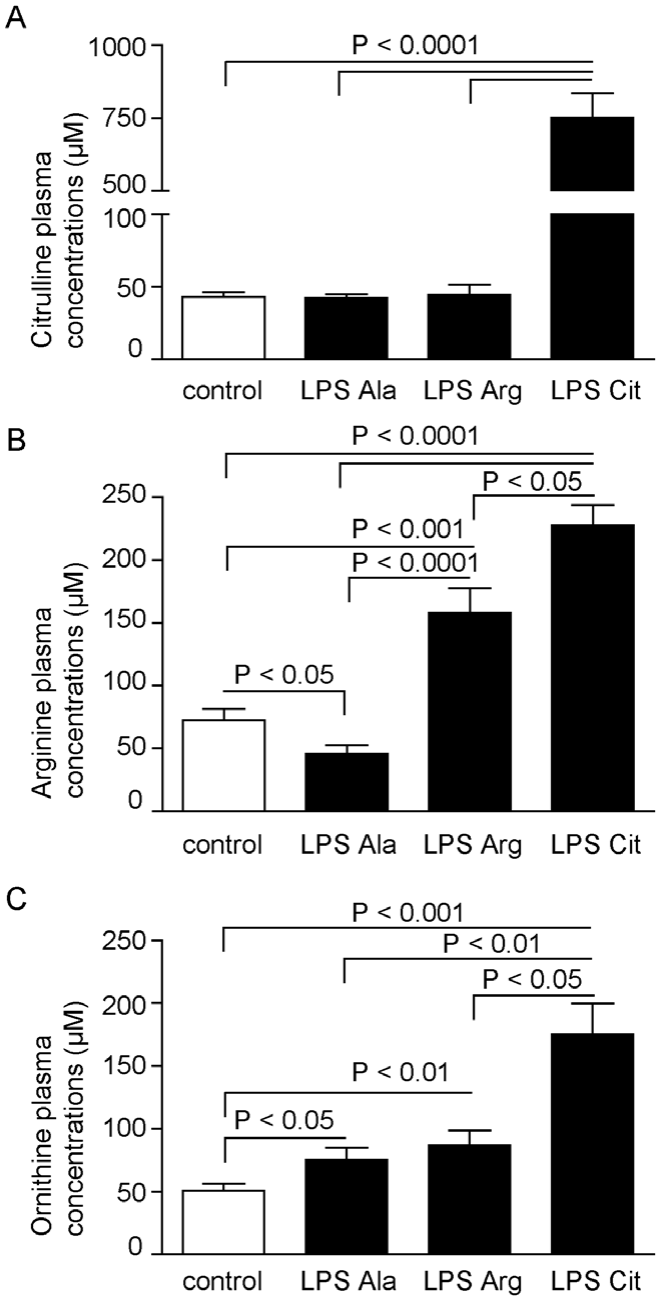
Amino-acid concentrations in plasma. (A) Citrulline plasma concentrations increased after L-Citrulline supplementation (LPS-Cit) compared to all other groups (P<0.0001). (B) Plasma arginine concentrations were significantly reduced in the LPS-Ala group compared to the control group (P<0.05), whereas this concentration was significantly increased by L-Arginine or L-Citrulline supplementation (LPS-Arg and LPS-Cit group) compared to both other groups (P<0.001). (C) Plasma ornithine levels increased in the LPS-Ala treated group compared to the control group (P<0.05) and increased further upon L-Arginine or L-Citrulline supplementation.

**Figure 5:**
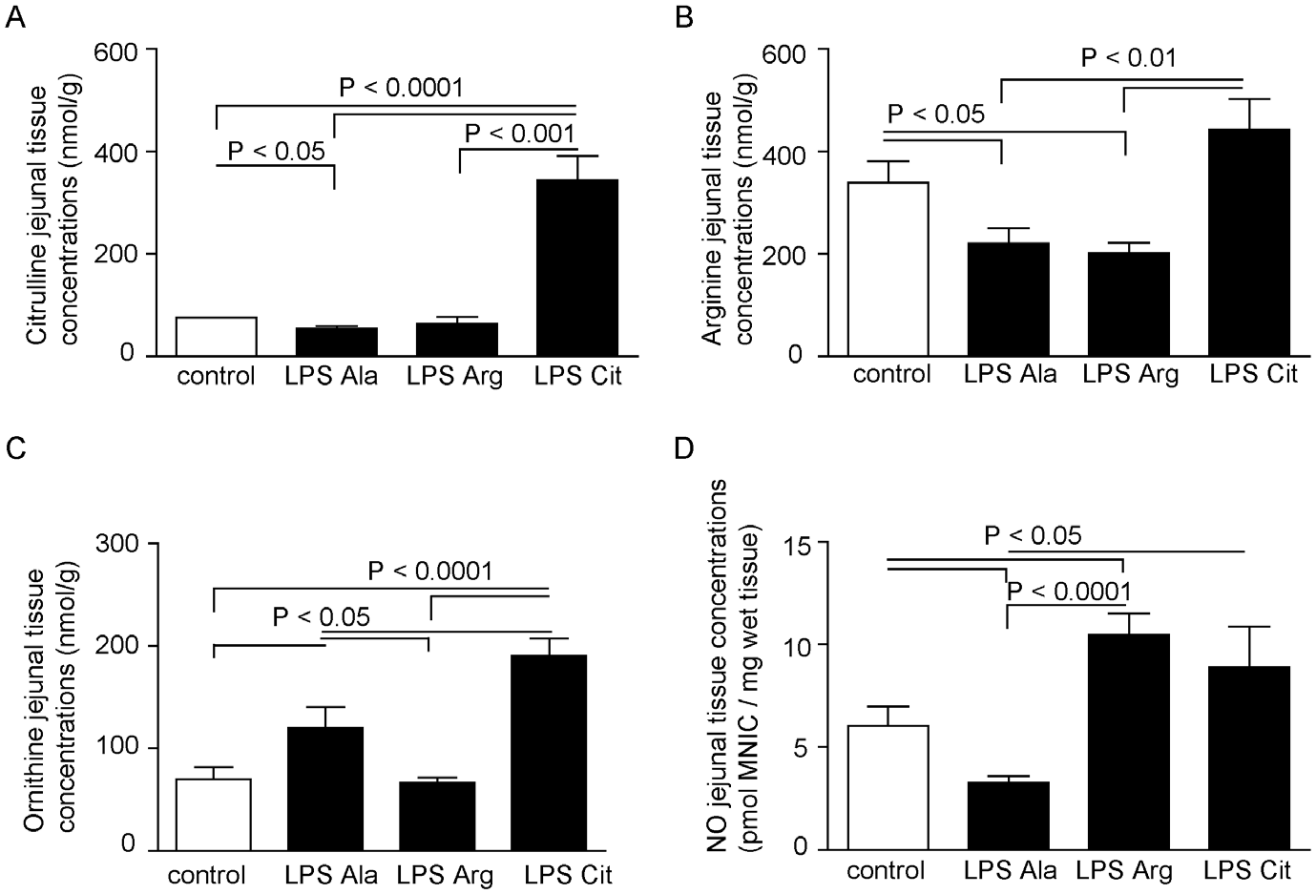
Amino-acid concentrations and NO production in murine jejunal tissue. (A) L-Citrulline supplementation (LPS-Cit) increased tissue citrulline concentration compared to the LPS-Ala, LPS-Arg and control groups (P<0.001) (B) Arginine concentrations were significantly reduced in the LPS-Ala group compared to the control group (P<0.05). Interestingly, this concentration was not increased by L-Arginine supplementation (LPS-Arg), whereas this concentration was significantly increased by L-Citrulline supplementation (LPS-Cit group; P<0.01). (C) Ornithine levels increased in the LPS-Ala treated group compared to the control group (P<0.05) and increased further upon L-Citrulline supplementation (P<0.0001 relative to the control group and P<0.05 relative to the LPS group). (D) In the jejunum, prolonged endotoxemia resulted in a significant decrease in NO production (measured as pmol mono-nitrosyl-iron complexes (MNIC)/mg wet tissue) compared to control (P<0.05). NO production was significantly improved in the LPS-Arg and LPS-Cit group compared to the LPS group (P<0.0001 and P<0.05 respectively).

**Figure 6:**
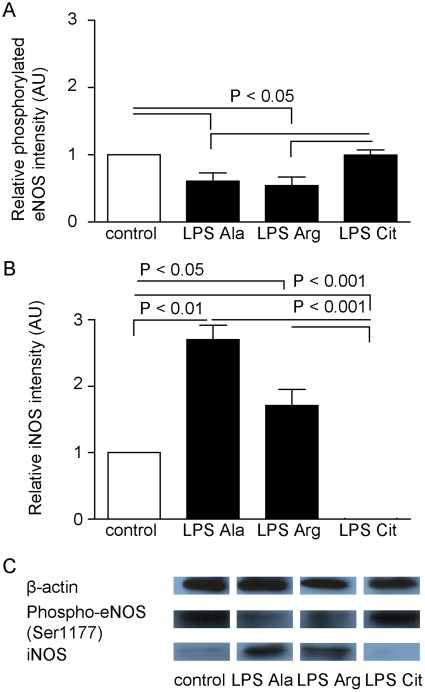
Phosphorylated eNOS and iNOS protein levels in murine jejunal tissue. (A) The degree of phosphorylation of eNOS protein (expressed in arbitrary units, AU) was higher in the LPS-Cit group than in the LPS-Ala and LPS-Arg group (P<0.05). (B) The iNOS protein concentration changed in an opposite detection, with significantly lower levels in the control (P<0.05) and LPS-Cit group (P<0.01) than in the LPS-Ala and LPS-Arg group. (C) Representative examples of expression of phosphorylated eNOS (Ser 1177), iNOS and beta-actin by Western blot analysis.

The mean arterial blood pressure (MAP) during these microcirculatory measurements was significantly higher in the control group versus the LPS-Ala group (control versus LPS-Ala, 70.1±1.8 versus 55.0±1.5 mmHg; P<0.01). In the L-Citrulline and L-Arginine supplemented groups the MAP was also significantly higher compared to the LPS-Ala group (LPS-Cit versus LPS-Ala, 64.7±1.5 versus 55.0±1.5 mmHg; P = 0.01 and LPS-Arg versus LPS-Ala, 65.0±2.8 versus 55.0±1.5 mmHg; P<0.05). The MAP was comparable between the LPS-Cit group and the LPS-Arg group (LPS-Cit versus LPS-Arg; P = 0.9). MAP did not differ significantly between the control group (70.1±1.8 mmHg) and the LPS-Cit or LPS-Arg group (P = 0.08 and P = 0.2, respectively).

### Citrulline Supplementation Improved the Microcirculation in the Gut During Endotoxemia

Endotoxemia resulted in a significantly decreased total number of perfused vessels (1 µm≤diameter≤50 µm) in the LPS-Ala group compared to control (n = 8; P<0.01; **Figure 2A**). L-Arginine administration during endotoxemia did not result in a restoration of the number of perfused vessels (n = 8; P<0.05). In contrast, L-Citrulline supplementation during endotoxemia restored the total number of perfused vessels to nearly the number of vessels seen in the control group (n = 8; P = 1.0). The observed difference in the total number of perfused vessels is caused by the decreased circulation in the smallest (≤10 µm) vessels in the LPS-Ala and LPS-Arg groups (42.5±6.5 and 33.7±4.1, respectively; **Figure 2B** **)**. The number of perfused microcirculatory vessels in the LPS-Cit group was comparable to that in control mice (Figure **2B**). Furthermore, also the total number as well as the number of small (≤10 µm) vessels perfused per villus were significantly decreased in the LPS-Ala and LPS-Arg group compared to the control and LPS-Cit treated group (Figure **2C and 2D** **)**. See **Figure 3A, 3B, 3C, 3D** and the **Videos S1**, **S2**, **S3, S4, S5** for representative live images and videos of the microcirculation in the respective groups. The characteristics of the microcirculation according to de Backer et al are displayed in the **Supporting Information Table S1**. Beneficial effects of L-Citrulline supplementation for these characteristics were found compared to the LPS-Ala and LPS-Arg group. L-Citrulline supplementation during physiological conditions did not influence the microcirculation (see **Supporting Information Table S1** and **Figure** **S2**).

### Citrulline Enhances Citrulline and Arginine Availability During Endotoxemia and Resulted in Increased Tissue NO Concentration

#### Plasma amino acids

As expected L-Citrulline supplementation increased plasma citrulline concentration ∼20-fold (P<0.0001) compared to the control, the LPS-Ala and the LPS-Arg group (**Figure 4A**). L-Citrulline supplementation during physiological conditions (NaCl-Cit group) did also result in enhanced plasma citrulline and arginine concentrations versus control (see **Figure S3**). Endotoxemia resulted in a significantly decreased arginine plasma concentration in the LPS-Ala group compared to control (n = 13; P<0.05), comparable to the metabolic disturbances in man. For a complete description of the L-arginine concentration during prolonged endotoxemia infusion see also **Figure S4**. In the LPS-Cit group the plasma arginine concentration was significantly increased versus control mice (n = 13; P<0.0001) and the LPS-Ala group (n = 13; P<0.0001). Although, L-Arginine supplementation enhanced the arginine plasma concentrations in the LPS-Arg group compared to the concentrations in control mice (n = 13; P<0.001; **Figure 4B**) and compared to LPS-Ala treated mice (n = 13; P<0.0001), these enhanced arginine levels were significantly lower than those in the L-Citrulline supplemented group (n = 13; P<0.05). The plasma ornithine concentration was higher in all LPS-treated groups than in the control group (**Figure 4C**).

#### Tissue amino-acid concentrations

The tissue amino-acid concentrations in all groups exhibited results that were largely similar to the changes in plasma concentrations. Endotoxemia resulted in a decreased jejunal tissue arginine concentration in the LPS-Ala group versus control (n = 13; P<0.05), which was accompanied by a significantly decreased tissue citrulline concentration (n = 13; P<0.05, **Figure 5A and 5B**). Interestingly, the beneficial effects of L-Arginine supplementation in the LPS-Arg group on the plasma concentrations was not observed in the jejunal tissue as the tissue arginine concentration was significantly decreased compared to control (n = 13; P<0.05, Figure 5A). However, L-Citrulline supplementation during endotoxemia also enhanced the tissue arginine concentration compared to LPS-Ala and LPS-Arg treated mice (n = 13; both P<0.01). L-Citrulline supplementation under basal conditions also enhanced the tissue arginine concentrations (see **Figure S3**). During endotoxemia ornithine tissue concentrations increased in the LPS-Ala and LPS-Cit treated groups compared to the control group, which was in line with the increase in the plasma concentrations (**Figure 5C**). In the LPS-Arg group ornithine tissue concentrations were lower than in the LPS-Ala group.

#### Tissue NO production

The jejunal NO production, detected as MNIC per mg jejunal tissue, decreased in the LPS-Ala group compared to the control group (n = 13, P<0.05). L-Arginine supplementation during endotoxemia enhanced the NO production compared to control and LPS-Ala treated animals (LPS-Arg versus control and versus LPS-Ala, n = 13; P<0.0001 and P<0.05, respectively; **Figure 5D**). Also L-Citrulline supplementation resulted in an increased NO production in the jejunum during basal and endotoxemic conditions compared to the LPS-Ala treated mice (n = 13; P<0.05; Figure 5D; basal data of the NaCl-Cit group see **Figure S5**).

### Citrulline Supplementation Affects the Expression Patterns of eNOS and iNOS in Endotoxemia

The extent of eNOS phosphorylation (Ser1177), as marker of active eNOS, in the jejunal tissue of the LPS-Ala and LPS-Arg treated groups was significantly decreased compared to the control group (both P<0.05). Interestingly, L-Citrulline supplementation to endotoxemic mice resulted in a higher jejunal degree of phosphorylation of eNOS than in the LPS-Ala, LPS-Arg or control mice (n = 6 in all groups, all P<0.05; **Figure 6A**). Moreover, a lower iNOS protein content was measured in the LPS-Cit group than in the LPS-Ala and LPS-Arg groups (n = 6) (**Figure 6B and 6C**: P<0.001). As expected the LPS-Ala group showed significantly increased iNOS protein levels compared to controls (n = 6) (**Figure 6B:** P<0.05).

## Discussion

Our study demonstrates beneficial effects of L-Citrulline supplementation in a mouse model of prolonged endotoxemia. We provide evidence for microcirculatory improvement with an increased number of perfused vessels in the microcirculation of the jejunal villi after L-Citrulline supplementation during endotoxemia, which was not observed following L-Arginine supplementation. These positive effects of citrulline appear to be mediated by increased plasma and tissue arginine availability, an enhanced presence of active phosphorylated eNOS and lower iNOS protein levels.

In this study we used a newly developed prolonged murine endotoxemia model (18 hours) to investigate the disturbances of the arginine-NO metabolism as reported in patients in whom prolonged exposure to endotoxin is expected. We, therefore, consider that this prolonged endotoxemia model mimics the human metabolic situation during endotoxemia-induced inflammatory conditions, in which we demonstrated a deficiency in arginine availability and associated decreased NO production during prolonged endotoxemia [44]. Thus far, disturbances in the arginine-NO metabolism were mainly studied in acute murine endotoxemia models [22], [45]–[48]. In these models, arginine as substrate for NO was identified as key factor in development of the microcirculatory and perfusion disturbances related to endotoxemia. However, no deficit in the plasma arginine pool, comparable to the metabolic derangements reported in man, was found in these acute models [22], [45]–[48]. It should however be mentioned that one experimental animal study reported significant changes in the arginine flux in the early stages of endotoxemia [48]. Our new model was based on a pilot observation in mice that plasma arginine concentrations decreased below baseline from 12 hours after the onset of the continuous endotoxin infusion, which is supported by the observations of Braulio et al [31]. This led us to use an 18 hours infusion period with endotoxin.

Arginine deficiency in inflammatory conditions has led to several experimental and human studies investigating modalities to restore the arginine-induced NO production [28]–[30], [49]. However, a study in critically ill patients supplemented with L-Arginine did not result in increased whole-body NO production, as assessed with the conversion of labeled arginine into citrulline using stable isotopic infusion, despite increased arginine plasma concentrations (Luiking, Poeze, Deutz, unpublished data). In addition, also in a model of prolonged porcine endotoxemia, whole-body NO production was not increased during L-Arginine supplementation [34]. Here we show that L-Arginine supplementation increased tissue NO production as assessed with ESR spectroscopy combined with *in vivo* NO-trapping, while the jejunal microcirculation did not improve. We consider that a disbalance between iNOS and eNOS-mediated NO release could explain the lack of improvement in microcirculation. Under normal physiological conditions, eNOS activity is central to the microcirculatory homeostasis. However, during endotoxemia and inflammation, iNOS is upregulated whereas eNOS expression is downregulated [25] resulting in a disrupted microvascular perfusion, a redistribution of the microcirculatory flow, hypoxia, and eventually organ failure [2], [25]. In line, the present study demonstrated a disturbed microcirculation and low degree in tissue phosphorylation of eNOS-protein levels in the LPS-Ala group, which were both not restored by supplementation of L-Arginine. Moreover, L-Arginine supplementation did not affect iNOS protein levels compared to the LPS-Ala group. Interestingly, L-Citrulline supplementation resulted in a similar tissue NO increase, while both arginine and citrulline tissue levels were increased. We consider that the reduced iNOS protein levels combined with the enhanced levels of active eNOS and the enhanced arginine concentrations in tissue following L-Citrulline supplementation are responsible for the improved microcirculation. These data strongly suggest that the citrulline availability appears to be the key factor rather than arginine to enhance the eNOS-derived NO production, to reduce iNOS protein levels and thereby improve organ perfusion during endotoxemia. This is supported by an in vitro study by Goodwin et al indicating intracellular arginine regeneration from citrulline, via the Ass pathway, to be essential for eNOS-mediated NO production in endothelial cells [50]. In line with these data, Shen et al. [27] reported that citrulline is the major arginine pool for eNOS-mediated NO production, while extracellular arginine forms the only arginine pool for iNOS-induced NO production [27]. In line, an enhanced NO production was reported to require not only increased circulating arginine concentrations, but also enhanced citrulline availability during endotoxemia [15]. The effects of L-Citrulline supplementation: enhanced plasma arginine concentration and increased tissue arginine concentration may indicate that insufficient citrulline availability underlies the low arginine levels seen in endotoxemic mice [15], [17], [22]. Such a therapeutic role of L-Citrulline supplementation is also supported by very recent data of Elwafi et al [36], who showed that oral supplementation of L-Citrulline in rats resulted in higher plasma arginine concentrations compared to L-Arginine supplementation during acute endotoxemia [36]. In addition, the therapeutic role of L-Citrulline is not limited to endotoxemic conditions, as L-Citrulline supplementation has been demonstrated to be beneficial in several other pathological conditions associated with limited L-Arginine availability or enhanced arginine catabolism [51]–[54]. In these (patho)physiological conditions, such as sickle cell disease, heart failure, and pulmonary hypertension, L-Citrulline was shown to be more efficient than L-Arginine to enhance the plasma levels of arginine and the eNOS-derived NO production. [52]–[58]. Therefore, L-Citrulline is a promising tool in raising arginine plasma concentrations and associated endothelial NO-production in diseases with arginine deficiency and vascular dysfunction.

In conclusion, this study demonstrates that L-Citrulline and not L-Arginine supplementation during murine endotoxemia improves the microcirculation and results in increased plasma and tissue availability of citrulline, arginine and NO during endotoxemia. The positive effects of L-Citrulline supplementation are paralleled by increased functional eNOS protein levels and reduced iNOS protein levels during endotoxemia.

## Supporting Information

Figure S1 **Evaluation of the endotoxemia signs in the LPS treated groups compared to the control group.** The average clinical signs of endotoxemia were more pronounced in the LPS-Ala and LPS-Arg group compared to control and LPS-Cit treated animals. The characteristic clinical manifestations of endotoxemia (Lethargy, hypothermia, diarrhea, piloerection/erection of the fur, exudates around the eyes and nostrils and diminished locomotor activity were scored by the animal care taker, blinded for treatment on a semi-quantitative 2 (absent/present) or 3-points (absent/moderate/severe) score. A 2-point score was used for lethargy, hypothermia and diarrhea and a 3-point score for piloerection, diminished locomotor activity and exudates around the eyes and nostrils.Data presented indicate median, interquartile, and 5%/95% range.
(TIF)

Figure S2 **Circulation measurements with SDF imaging in the NaCl-Cit group compared to control.** (A) L-Citrulline supplementation during physiological conditions did not enhance the total number (mean±SEM) of measurable perfused vessels in the jejunal microcirculation, (B) or the vessels with a diameter ≤10 µm, compared to control. (C) Also at the villus level, L-Citrulline supplementation did not enhance the total number of measurable perfused vessels. (D) A distribution of the number of perfused vessels per vessel diameter (µm) shows a comparable number of vessels per group. (E) Also the vessels with a diameter ≤10 µm per villus, did not differ between groups.
(TIF)

Figure S3 **Plasma and jejunal tissue amino-acid concentrations under physiological conditions with or without L-Citrulline supplementation.** (A) Citrulline, arginine and ornithine plasma concentrations increased after L-Citrulline supplementation NaCl-Cit) compared to control (P<0.001). (B) Tissue amino-acid concentrations of citrulline, arginine and ornithine were also significantly increased by L-Citrulline supplementation under physiological conditions.
(TIF)

Figure S4 **Time-course of plasma arginine concentrations during prolonged LPS infusion.** Arginine plasma concentrations initially increased during the first hours of prolonged endotoxemia. However, arginine plasma concentrations decreased significantly below basal concentrations after 12 hours of continuous LPS infusion, which indicated an arginine deficient state in plasma. Each time point contains plasma concentrations of 6 mice. Data represented in mean ± SEM.
(TIF)

Figure S5 **Intracellular NO production in murine jejunal tissue under physiological conditions with or without L-Citrulline supplementation.** Citrulline supplementation (NaCl-Cit) did not result in enhanced NO production (measured as pmol mono-nitrosyl-iron complexes (MNIC)/mg wet tissue weight) compared to control in murine jejunal tissue.
(TIF)

Table S1 **Summary of microcirculatory parameters in control groups and the LPS-treated groups with/without citrulline or arginine supplementation.** Total vessel density (TVD; mm/mm^2^) was calculated as the number of vessels crossing the arbitrary lines in Ava 3.0 divided by the total length of the lines. The perfused vessel density (PVD; mm/mm^2^) and proportion of perfused vessel (PPV; %) were calculated based on the TVD and perfusion of the total vessels. The microvascular flow index (MFI) is based on the determination of the predominant type of flow in the repeating microvascular structure (villi) in the four quadrants of the image (0 = absent, 1 = intermittent, with at least 50% of the time no flow, 2 = sludging, 3 = normal or 4 = hyperdynamic flow). The percentage of perfused villi was calculated as the number of perfused villi divided by the total number of villi present in the high power field. Superscript ^a^ P-value <0.05 comparing the LPS-Cit with the LPS-Ala group. Superscript ^b^ P-value <0.01 comparing LPS-Cit with the LPS-Arg group. Superscript ^c^ P-value <0.05 comparing the control with the LPS-Arg group. Superscript ^d^ P-value <0.01 comparing the NaCl-Cit with the LPS-Arg group. Superscript ^e^ P-value <0.0001 comparing the control with the LPS-Ala group. Superscript ^f^ P-value <0.01 comparing the control with the LPS-Arg group. Superscript ^g^ P-value <0.0001 comparing the NaCl-Cit with the LPS-Ala group. Superscript ^h^ P-value <0.01 comparing the NaCl-Cit with the LPS-Ala group. Superscript ^i^ P-value <0.05 comparing LPS-Cit with the LPS-Arg group. Superscript ^j^ P-value <0.01 comparing control with the LPS-Ala group. Data are shown as mean ± SEM. Statistical significance was determined with one-way ANOVA with post-hoc Bonferroni correction between groups.
(DOC)

Methods S1 Detailed information of material and methods used in this study.
(DOC)

Video S1 **Live imaging with SDF imager of the jejunal microcirculation in the villi of control mouse.** In this video file the microcirculation is equally distributed in the villi, with a continuous and homogeneous distribution of the perfusion in the small and large vessels.
(AVI)

Video S2 **Live imaging with SDF imager of the jejunal microcirculation in the villi of LPS-Ala treated mouse.** The video shows a decreased perfusion of the microcirculation with flow mainly persisting in the largest vessels.
(AVI)

Video S3 **Live imaging with SDF imager of the jejunal microcirculation in the villi of LPS-Arg treated mouse.** The video shows a comparable decreased perfusion of the microcirculation as seen in the LPS-Ala treated mouse, with flow mainly persisting in the largest vessels.
(AVI)

Video S4 **Live imaging with SDF imager of the jejunal microcirculation in the villi of LPS–Cit treated mouse.** In this video file, the smallest microcirculatory vessels are also perfused. Furthermore, the perfusion is continuous and homogeneously distributed.
(AVI)

Video S5 **Live imaging with SDF imager of the jejunal microcirculation in the villi of NaCl-Cit treated mouse.** In this video the microcirculatory distribution is comparable to that in the control group with a continuous and homogeneously distributed perfusion.
(AVI)
