# Supplementary figures and images for: Citrulline a More Suitable Substrate than Arginine to Restore NO Production and the Microcirculation during Endotoxemia
Source: PLoS One. 2012 May 29;7(5):e37439. doi: 10.1371/journal.pone.0037439 (PMC3362574; doi:10.1371/journal.pone.0037439)

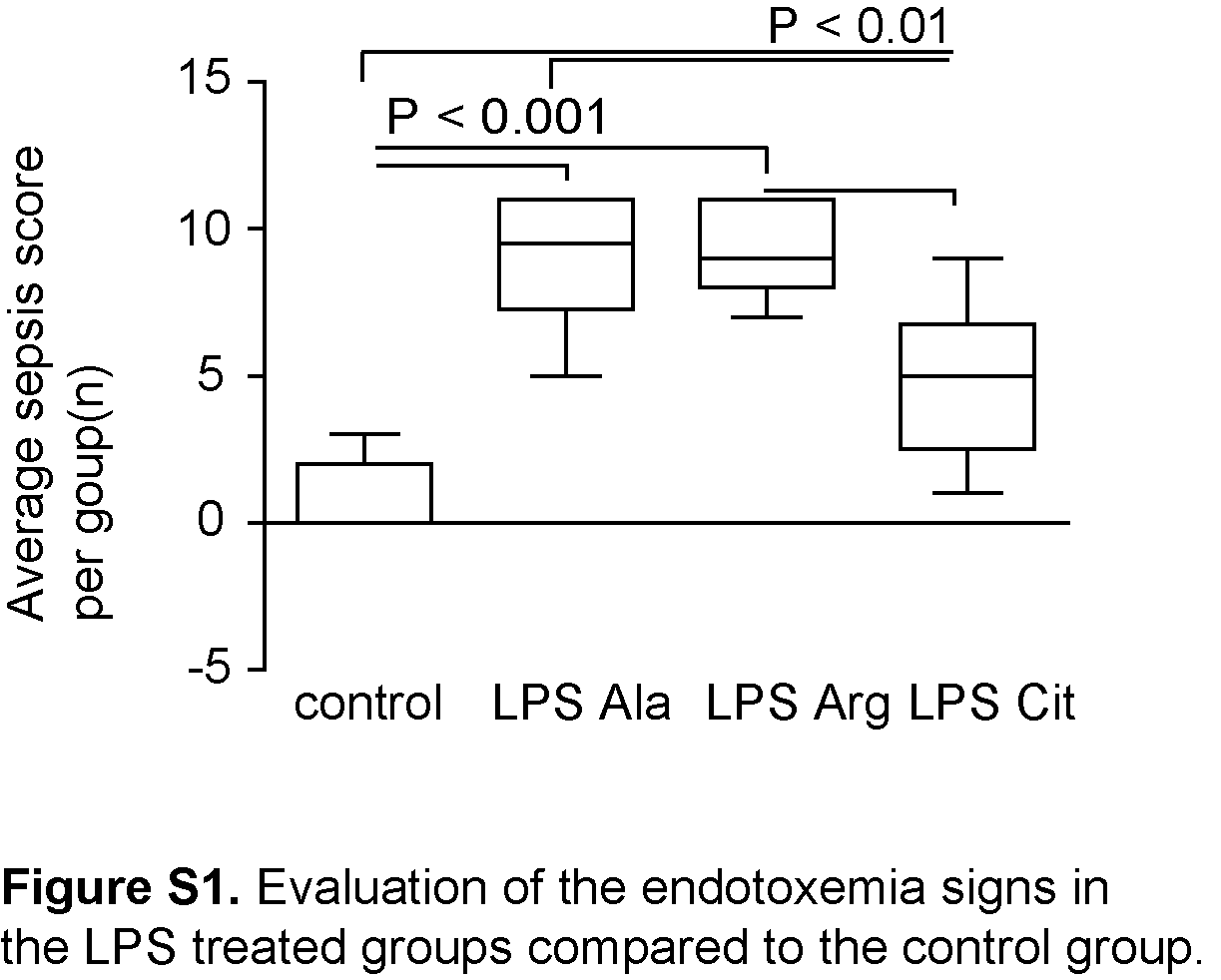

Supplement: Figure S1 — Evaluation of the endotoxemia signs in the LPS treated groups compared to the control group. The average clinical signs of endotoxemia were more pronounced in the LPS-Ala and LPS-Arg group compared to control and LPS-Cit treated animals. The characteristic clinical manifestations of endotoxemia (Lethargy, hypothermia, diarrhea, piloerection/erection of the fur, exudates around the eyes and nostrils and diminished locomotor activity were scored by the animal care taker, blinded for treatment on a semi-quantitative 2 (absent/present) or 3-points (absent/moderate/severe) score. A 2-point score was used for lethargy, hypothermia and diarrhea and a 3-point score for piloerection, diminished locomotor activity and exudates around the eyes and nostrils.Data presented indicate median, interquartile, and 5%/95% range. (TIF) [file pone.0037439.s001.tif]

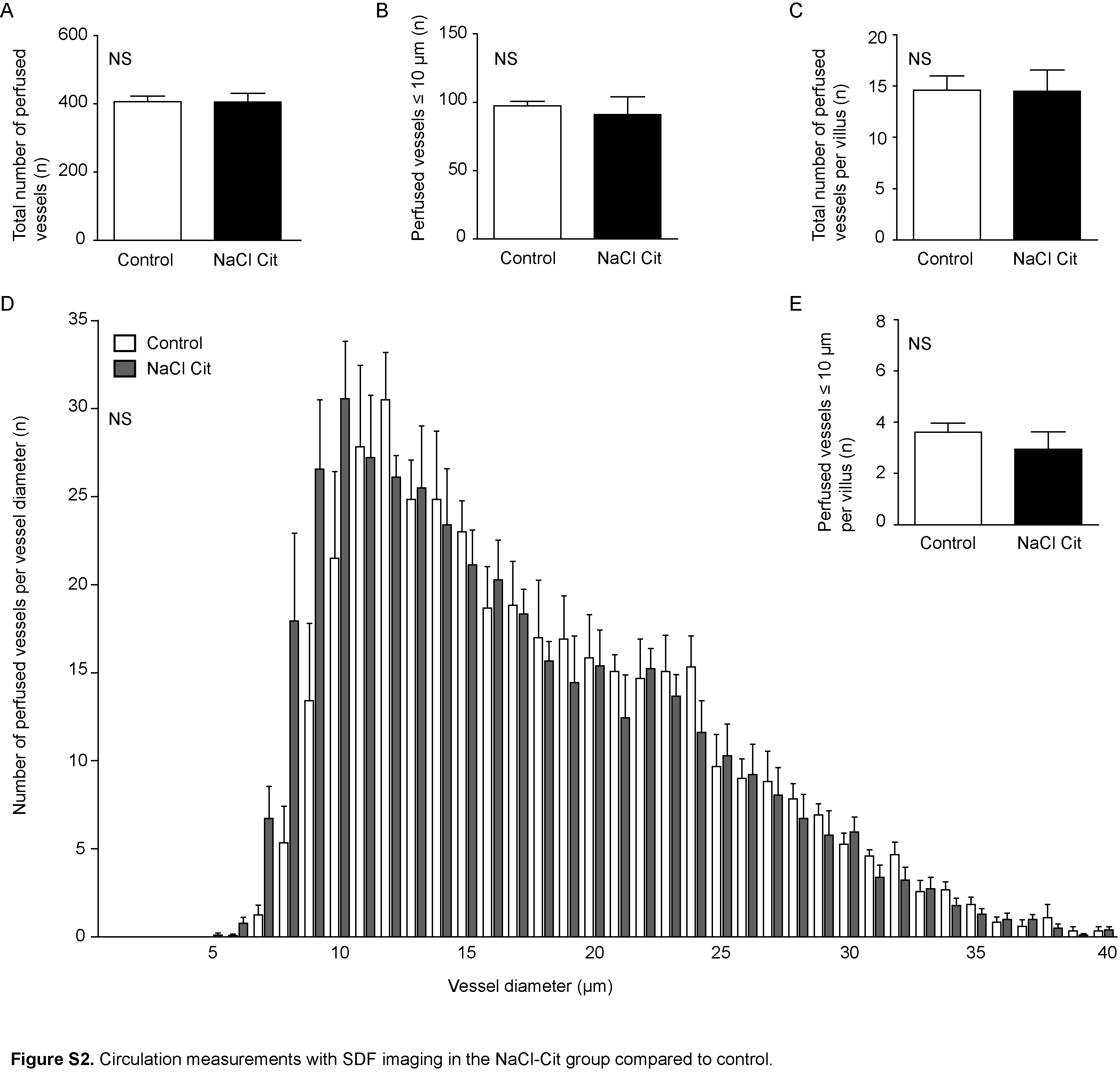

Supplement: Figure S2 — Circulation measurements with SDF imaging in the NaCl-Cit group compared to control. (A) L-Citrulline supplementation during physiological conditions did not enhance the total number (mean±SEM) of measurable perfused vessels in the jejunal microcirculation, (B) or the vessels with a diameter ≤10 µm, compared to control. (C) Also at the villus level, L-Citrulline supplementation did not enhance the total number of measurable perfused vessels. (D) A distribution of the number of perfused vessels per vessel diameter (µm) shows a comparable number of vessels per group. (E) Also the vessels with a diameter ≤10 µm per villus, did not differ between groups. (TIF) [file pone.0037439.s002.tif]

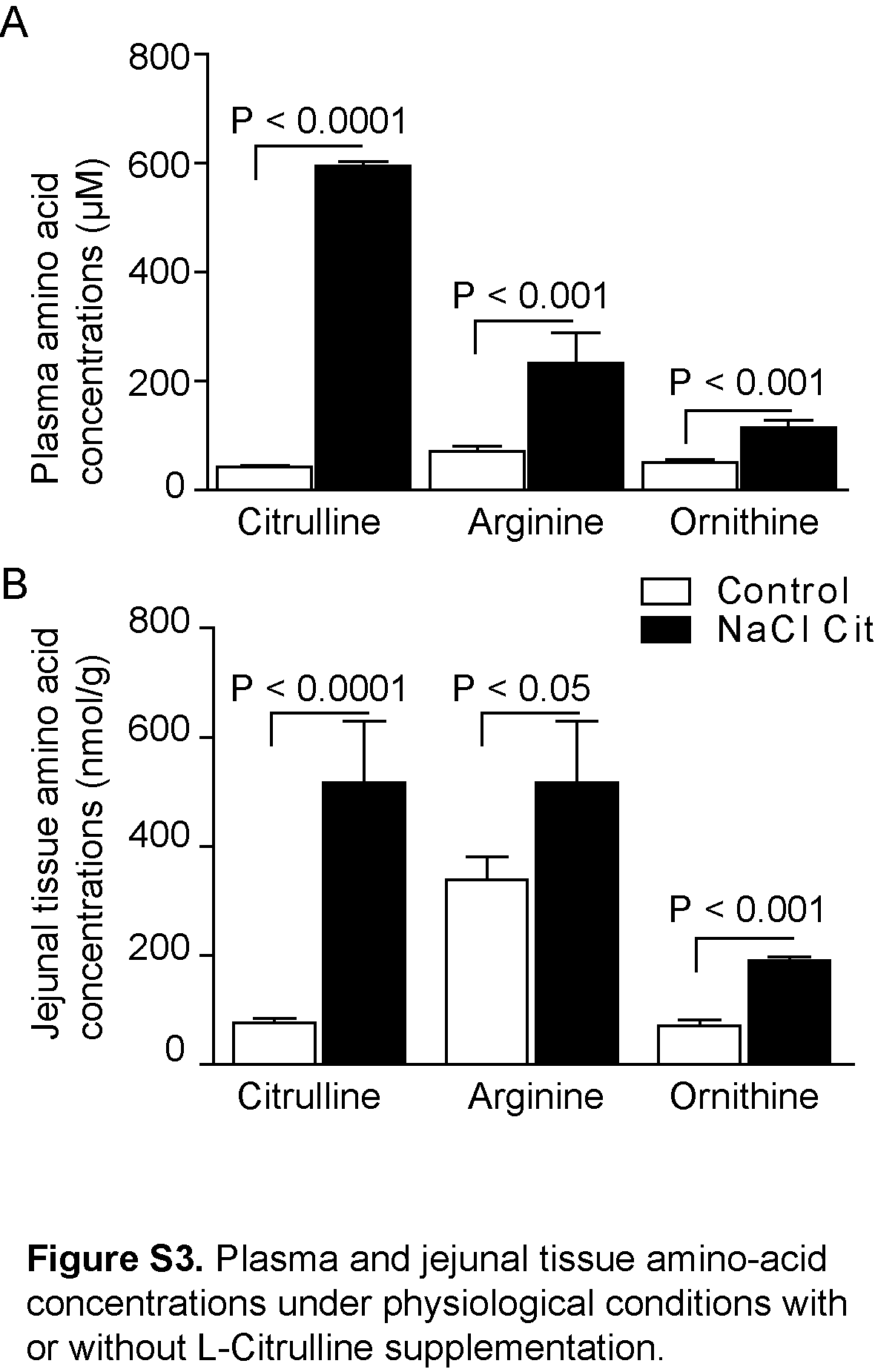

Supplement: Figure S3 — Plasma and jejunal tissue amino-acid concentrations under physiological conditions with or without L-Citrulline supplementation. (A) Citrulline, arginine and ornithine plasma concentrations increased after L-Citrulline supplementation NaCl-Cit) compared to control (P<0.001). (B) Tissue amino-acid concentrations of citrulline, arginine and ornithine were also significantly increased by L-Citrulline supplementation under physiological conditions. (TIF) [file pone.0037439.s003.tif]

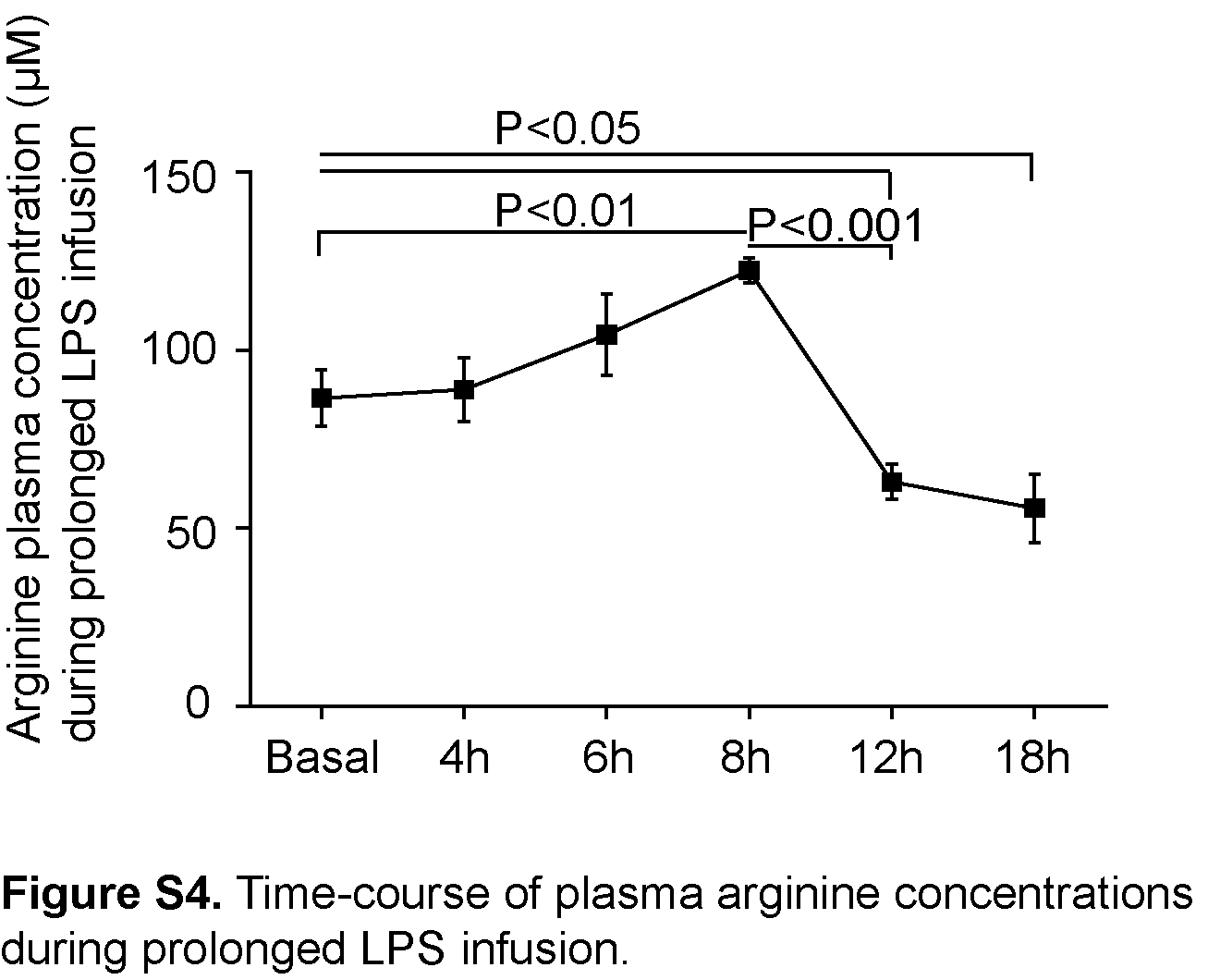

Supplement: Figure S4 — Time-course of plasma arginine concentrations during prolonged LPS infusion. Arginine plasma concentrations initially increased during the first hours of prolonged endotoxemia. However, arginine plasma concentrations decreased significantly below basal concentrations after 12 hours of continuous LPS infusion, which indicated an arginine deficient state in plasma. Each time point contains plasma concentrations of 6 mice. Data represented in mean ± SEM. (TIF) [file pone.0037439.s004.tif]

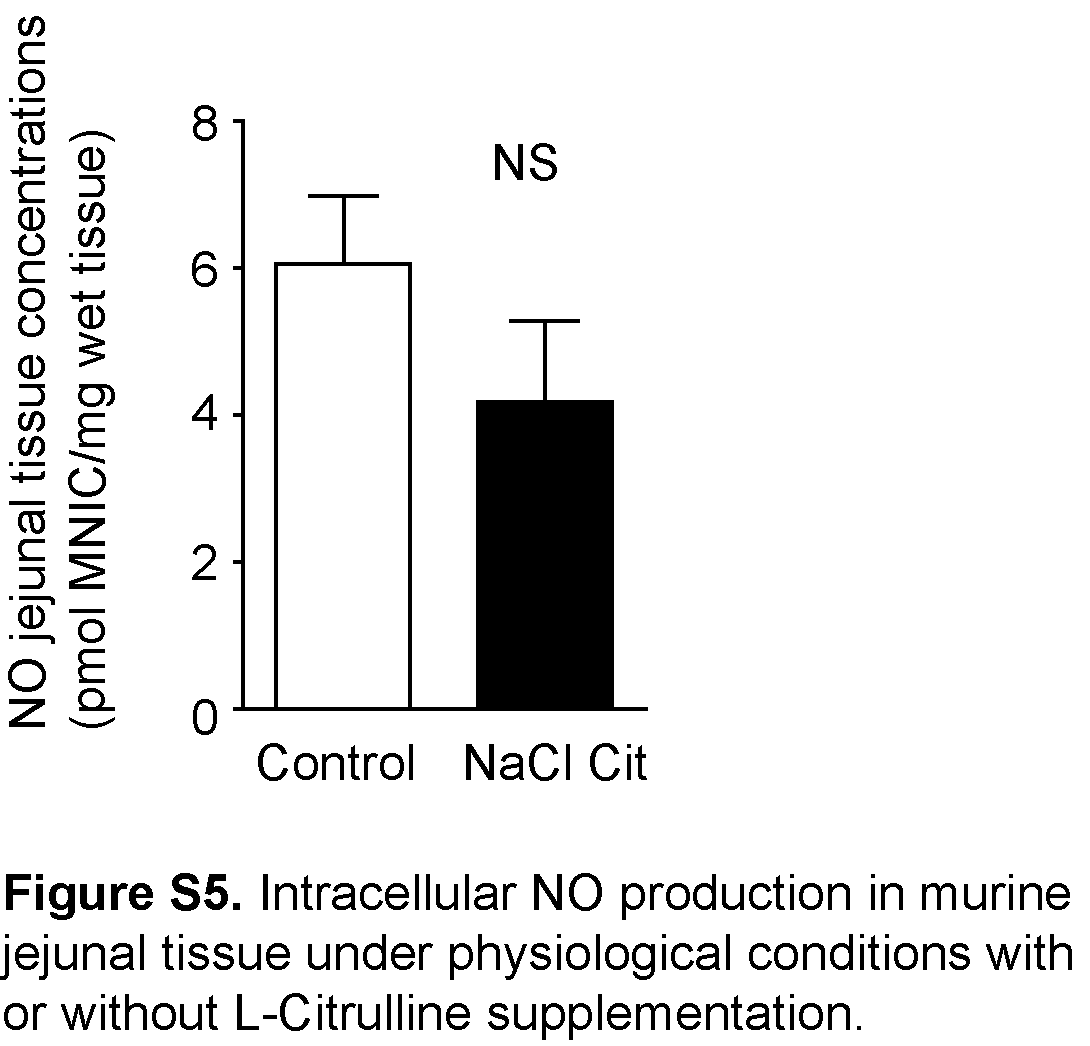

Supplement: Figure S5 — Intracellular NO production in murine jejunal tissue under physiological conditions with or without L-Citrulline supplementation. Citrulline supplementation (NaCl-Cit) did not result in enhanced NO production (measured as pmol mono-nitrosyl-iron complexes (MNIC)/mg wet tissue weight) compared to control in murine jejunal tissue. (TIF) [file pone.0037439.s005.tif]
