# Supplementary material for: Citrulline a More Suitable Substrate than Arginine to Restore NO Production and the Microcirculation during Endotoxemia
Source: PLoS One. 2012 May 29;7(5):e37439. doi: 10.1371/journal.pone.0037439 (PMC3362574; doi:10.1371/journal.pone.0037439)
Supplement: Table S1 — Summary of microcirculatory parameters in control groups and the LPS-treated groups with/without citrulline or arginine supplementation. Total vessel density (TVD; mm/mm2) was calculated as the number of vessels crossing the arbitrary lines in Ava 3.0 divided by the total length of the lines. The perfused vessel density (PVD; mm/mm2) and proportion of perfused vessel (PPV; %) were calculated based on the TVD and perfusion of the total vessels. The microvascular flow index (MFI) is based on the determination of the predominant type of flow in the repeating microvascular structure (villi) in the four quadrants of the image (0 = absent, 1 = intermittent, with at least 50% of the time no flow, 2 = sludging, 3 = normal or 4 = hyperdynamic flow). The percentage of perfused villi was calculated as the number of perfused villi divided by the total number of villi present in the high power field. Superscript a P-value <0.05 comparing the LPS-Cit with the LPS-Ala group. Superscript b P-value <0.01 comparing LPS-Cit with the LPS-Arg group. Superscript c P-value <0.05 comparing the control with the LPS-Arg group. Superscript d P-value <0.01 comparing the NaCl-Cit with the LPS-Arg group. Superscript e P-value <0.0001 comparing the control with the LPS-Ala group. Superscript f P-value <0.01 comparing the control with the LPS-Arg group. Superscript g P-value <0.0001 comparing the NaCl-Cit with the LPS-Ala group. Superscript h P-value <0.01 comparing the NaCl-Cit with the LPS-Ala group. Superscript i P-value <0.05 comparing LPS-Cit with the LPS-Arg group. Superscript j P-value <0.01 comparing control with the LPS-Ala group. Data are shown as mean ± SEM. Statistical significance was determined with one-way ANOVA with post-hoc Bonferroni correction between groups. (DOC) [file pone.0037439.s006.doc]

**Supporting Information Table S1. Summary of microcirculatory parameters in control groups and the LPS-treated groups with/without citrulline or arginine supplementation**.

|  | **Control**  **(n=8)** | **NaCl-Cit**  **(n=8)** | **LPS-Ala**  **(n=8)** | **LPS-Arg**  **(n=8)** | **LPS-Cit**  **(n=8)** |
| --- | --- | --- | --- | --- | --- |
| **Density** |  |  |  |  |  |
| **TVD** (mm/mm^2^) | 26.0±2.2 | 27.3±0.6 | 21.1±1.8^a^ | 19.0±1.1^b,c,d^ | 27.7±1.1 |
| **PVD** (mm/mm^2^) | 26.0±2.2 | 27.3±0.6 | 19.5±2.7^a^ | 18.3±1.5^b,c,d^ | 27.7±1.1 |
| **Perfusion** |  |  |  |  |  |
| **PPV** (%) | 100±0.0 | 100±0.0 | 91.7±8.3 | 98.2±0.9 | 100±0.0 |
| **MFI (**Flow villi) | 3.0±0.0 | 3.0±0.0 | 1.9±0.2^a,e,g^ | 2.1±0.1^d,f,h^ | 2.9±0.1 |
| **Perfused villi**(%) | 100±0.0 | 100±0.0 | 66.3±4.3^a,j^ | 65.2±4.6^f,i^ | 97.8±1.2 |

Total vessel density (TVD; mm/mm^2^) was calculated as the number of vessels crossing the arbitrary lines in Ava 3.0 divided by the total length of the lines. The perfused vessel density (PVD; mm/mm^2^) and proportion of perfused vessel (PPV; %) were calculated based on the TVD and perfusion of the total vessels. The microvascular flow index (MFI) is based on the determination of the predominant type of flow in the repeating microvascular structure (villi) in the four quadrants of the image (0 = absent, 1 = intermittent, with at least 50% of the time no flow, 2 = sludging, 3 = normal or 4 = hyperdynamic flow). The percentage of perfused villi was calculated as the number of perfused villi divided by the total number of villi present in the high power field. Superscript ^a^ P-value < 0.05 comparing the LPS-Cit with the LPS-Ala group. Superscript ^b^ P-value < 0.01 comparing LPS-Cit with the LPS-Arg group. Superscript ^c^ P-value < 0.05 comparing the control with the LPS-Arg group. Superscript ^d^ P-value < 0.01 comparing the NaCl-Cit with the LPS-Arg group. Superscript ^e^ P-value < 0.0001 comparing the control with the LPS-Ala group. Superscript ^f^ P-value < 0.01 comparing the control with the LPS-Arg group. Superscript ^g^ P-value < 0.0001 comparing the NaCl-Cit with the LPS-Ala group. Superscript ^h^ P-value < 0.01 comparing the NaCl-Cit with the LPS-Ala group. Superscript ^i^ P-value < 0.05 comparing LPS-Cit with the LPS-Arg group. Superscript ^j^ P-value < 0.01 comparing control with the LPS-Ala group. Data are shown as mean ± SEM. Statistical significance was determined with one-way ANOVA with post-hoc Bonferroni correction between groups.
