# Supplementary material for: Citrulline a More Suitable Substrate than Arginine to Restore NO Production and the Microcirculation during Endotoxemia
Source: PLoS One. 2012 May 29;7(5):e37439. doi: 10.1371/journal.pone.0037439 (PMC3362574; doi:10.1371/journal.pone.0037439)
Supplement: Methods S1 — Detailed information of material and methods used in this study. (DOC) [file pone.0037439.s007.doc]

# Citrulline a More Suitable Substrate than Arginine to Restore NO Production and the Microcirculation during Endotoxemia

Karolina AP Wijnands, MD; Hans Vink, PhD; Jacob J Briedé, PhD; Ernst E van Faassen, PhD; Wouter H Lamers, MD, PhD; Wim A Buurman, PhD; Martijn Poeze, MD, PhD

**ON LINE Supporting Information**

**Supporting Information methods**

**Animals**

Mice were individually housed and subject to standard 12 hour light-dark cycle periods. The mice were fed standard lab chow (Hope Pharms, Woerden, the Netherlands) and water *ad libitum* until the final stage of the experiment. Room temperature was maintained at 25°C. Food intake was measured daily by comparing the quantity of chow in the cage to the quantity given the day before. The mice were weighed daily to monitor post-operative weight gain.

**Surgical procedure**

After the mice were weighed and premedicated with 0.01mg/kg Temgesic® (Reckitt & Colman Products LTD., Kingston-Upon Hill, England) subcutaneously, anesthesia was induced with 4% Isoflurane (Abbott Laboratories LTD, England). During surgery anesthesia was maintained with 2% Isoflurane. Throughout the experiment, body temperature was maintained at 37°C, using an infrared heating lamp with a temperature controller connected to a rectal probe. Fluid resuscitation was provided prior to the surgical intervention with a single subcutaneous warm sterile 0.9% saline injection (1.5mL).

A longitudinal incision (0.5cm) was made on top of the head to expose the skull for fixation of a cannula. Prior to removing the periosteum, a drop of 1% lidocaine was applied for topical analgesia. A longitudinal incision (1.5cm) in the neck exposed the area of the right jugular vein. The catheter (0.020”x0.037” Silclear tubing, MEDNET, Münster, Germany) was filled with heparinized saline, tunneled subcutaneously to the skull and inserted in the right jugular vein. A 30-gauge needle, bent in a 90º angle, was fixed with glass ionomer cement (FuijCEM Automix, Instech, Solomon Plymouth Meeting, PA) to the skull and used to close the catheter. A head block was attached simultaneously with the catheter to connect the swivel during experiments. The skin was closed with Softsilk 4.0 sutures (Covidien, Norwalk, CT). Rimadyl® (Pfizer Inc., NY, 5 mg/kg) and Temgesic® (0.05mg/kg) were subcutaneously administered for post-operative analgesia. The mice remained in an incubator at 37ºC during the first 2 post-operative hours to recover from surgery.

**Experimental protocol**

The experiment started 4 days after the initial cannulation. During the experimental period, the mice were deprived of food. The mice were attached to a swivel system for continuous infusion and randomly allocated, in a non-blinded fashion, to either an 18hour sterile 0.9% saline (n=26) or lipopolysaccharide (LPS) endotoxin infusion (E.Coli O55:B5, Sigma Aldrich, St.Louis, MO) (n=39). LPS (0.4µg•g body weight^-1^•h^-1^) was infused during the 18hour period with a continuous flow rate of 83 μL/h and 1.5mL fluid in total. In the final 6 hours of the LPS infusion period, L-Citrulline (LPS-Cit; 6.25mg/h), L-Arginine (LPS-Arg; 6.25mg/h) or an isonitrogenous concentration of the placebo amino acid L-Alanine (LPS-Ala; 12.5mg/h) was added. The control group was treated with 0.9% saline and L-Alanine only (Control n=13). To investigate the role of L-Citrulline in physiological conditions a group supplemented with sterile saline and L-Citrulline (NaCl-Cit group n=13) was investigated.

At the end of the infusion period, anesthesia was induced as described above for a second surgical procedure. A small segment of the jejunum was exteriorized via a 0.5cm incision in the midline. Loperamidehydrochloride (2.5mg/mL, Marel BV Leiden, the Netherlands) was administered into the jejunal segment to decrease the intestinal motility, which was comparable between groups. A longitudinal incision (0.5cm) in the jejunal segment was made to visualise the inner surface of the intestine microscopically with the sidestream dark-field (SDF) imager (Microscan, Amsterdam, The Netherlands). During surgery anesthesia was maintained with 2% Isoflurane while body temperature was maintained as described above. At the end of the experiment the mouse was sacrificed through a cardiac puncture for blood sampling. During the endotoxin infusion the condition of the mice was assessed using a predefined score of clinical features of endotoxemia [1]. No animals died during the experiments.

**Microcirculation measurements**

SDF imaging was used to discriminate between vessels of different diameter and to assess the proportion of vessels in the jejunal villi that were perfused. The SDF imager uses 530nm light absorbed by the haemoglobin in red blood cells which allows observation of these cells in the microcirculation [2]. All imaging experiments were done by an experienced investigator. Using a camera magnification of 5x, sharp real-time images of the microcirculation in the jejunal villi were obtained in a field of 1000x750μm. In total 20-sec continuous image sequences per mouse, each consisting of 200 images was recorded at five sites. According to the consensus of a round table conference [3], a minimum of 3-5 sites of the jejunum per animal should be analyzed and the images of poor quality should be discarded from analysis. This resulted in 3-5 sites per animal which could be reliably evaluated. In total 166 videos were analyzed of 16 control (8 control and 8 NaCl-Cit), each with 40 videos per group, and 24 animals receiving endotoxemia (8 each for LPS-Ala, LPS-Arg and LPS-Cit). In the LPS-Ala group 28 videos were analyzed, in the LPS-Arg group 27 videos and 31 videos in the LPS-Cit group. Fragments of 60 images per time point per site were selected for analysis with Image J (version1.43, downloaded from www.rsb.info.nih.gov/ij/download) and Matlab to evaluate the vessel diameter and the number of perfused vessels. Prior to manual identification of the blood vessels and the calculation of vascular density, linear transformation was used for calibration and image stabilisation. Videos were analyzed by 2 independent researchers according to de Backer et al. [3].

**In vivo tissue NO measurements**

To determine the in vivo NO production in tissues spin trap agents were administered to 5 mice per group [4,5]. Mice were injected subcutaneously in the scruff of the neck with a mixture of FeSO_4_·7H_2_O (37.5mg/kg), sodium citrate (190mg/kg) and intraperitoneally with diethyldithiocarbamate (DETC) 500mg/kg 30 minutes prior to sacrifice. The chemicals, all purchased from Sigma-Aldrich**,** were dissolved in 0.1 ml HEPES buffer (15mM, pH 7.4). During NO spintrapping, the hydrophobic Fe^2+^-(DETC)_2_ complexes are immobilized in the low-polarity lipid fraction or protein fraction of the tissue. The Fe^2+^-(DETC)_2_ complexes trap all the free NO radicals inside and outside the cell, as this complex is associated with the cell membranes, thereby forming stable paramagnetic mononitrosyl-iron complexes (MNIC) which accumulate in the tissue within time [6]. After NO spintrapping for 30 min, mice were anesthesized with Isoflurane. The abdominal cavity opened and jejunum collected for ESR quantification of the MNIC content in the tissues, after flushing the afferent artery with normal saline. Approximately 100-200mg of tissue was transferred to a plastic syringe (diameter 4.8mm) filled with HEPES buffer (150mM, pH 7.4) and snap frozen in liquid nitrogen. Samples were stored at -80ºC until analysis with electron spin resonance (ESR) spectroscopy.

To reduce possible influence of the Cu^2+^DETC on the NO triplet, the sample was thawed and incubated with solid sodium dithionite (50mM) for 15 minutes at room temperature, prior to snap-freezing again and before NO-signal determination with Electron Spin-Resonance (EPR). The frozen samples were placed in a quartz liquid finger Dewar at the center of the 1273 ER4119HS high sensitivity cavity. The EPR spectra of NO were recorded on an X-band spectrometer (Bruker EMX 1273, Biospin, Rheinstetten, Germany) operating at 9.43GHz with a 20mW microwave power. The magnetic field was 100kHz with a 5G amplitude. NO concentrations were calculated from the height of three line NO spectrum with Bruker WINEPR software as described [4,7].

**Protein isolation and Western blot analysis**

Protein was isolated using the AllPrep DNA/RNA/Protein kit (Qiagen, Hilden, Germany) according to the manufacturer’s protocol. Jejunal samples were crushed in liquid nitrogen with a pestle and mortar, and disrupted and homogenized with the Ultra Turrax Homogenizer (IKA, Labortechnik, Staufen, Germany) in lysis buffer containing β-mercapto-ethanol (Promega, Madison, WI). Proteins were precipitated in the flow-through of the RNeasy spin column. The protein precipitate was centrifuged and its pellet dissolved in 5% SDS (sodium dodecyl sulfate). Samples were stored at -80°C until analysis.

Sample protein concentrations were determined using a Microplate BCA protein assay kit (Pierce, Etten-Leur, The Netherlands). Prior to loading of 10µg of the total protein per sample onto a 10% polyacrylamide gel, samples were incubated at 95°C in SDS sample buffer containing β-mercapto-ethanol to completely dissolve and denature the protein. Polyvinylidene-fluoride membranes (ImmobiliP, Millipore, Bredford, MA) were used for blotting. Membranes were blocked in 3% milk solution and incubated overnight with rabbit polyclonal anti-mouse iNOS (Abcam, Cambridge, MA) or rabbit polyclonal anti-mouse phosphorylated eNOS (SER 1177) (Cell signaling technology, Danvers, MA) at 4**°**C. After washing, membranes were incubated with HRP-conjugated goat anti-rabbit secondary antibody for iNOS and phosphorylated eNOS (Jackson Immunoresearch Laboratories, West Grove, Pennsylvania, USA). Membranes were re-probed with anti-mouse β-actin (Sigma) and HRP-conjugated rat anti-mouse secondary antibody (Jackson) to demonstrate equal loading and transfer of the samples. A chemiluminescence reaction with SuperSignal West Pico Chemiluminescent Substrate (Pierce) was used to capture the signals on X-ray film (Fuji SuperRX, Tokyo, Japan).

**Supporting information references**

1. Olfert ED, Godson DL (2000) Humane endpoints for infectious disease animal models. Ilar J 41: 99-104.

2. Boerma EC, Mathura KR, van der Voort PH, Spronk PE, Ince C (2005) Quantifying bedside-derived imaging of microcirculatory abnormalities in septic patients: a prospective validation study. Crit Care 9: R601-606.

3. De Backer D, Hollenberg S, Boerma C, Goedhart P, Buchele G, et al. (2007) How to evaluate the microcirculation: report of a round table conference. Crit Care 11: R101.

4. van Faassen EE, Koeners MP, Joles JA, Vanin AF (2008) Detection of basal NO production in rat tissues using iron-dithiocarbamate complexes. Nitric Oxide 18: 279-286.

5. Sjakste N, Andrianov VG, Boucher JL, Shestakova I, Baumane L, et al. (2007) Paradoxical effects of two oximes on nitric oxide production by purified NO synthases, in cell culture and in animals. Nitric Oxide 17: 107-114.

6. Kleschyov AL, Wenzel P, Munzel T (2007) Electron paramagnetic resonance (EPR) spin trapping of biological nitric oxide. J Chromatogr B Analyt Technol Biomed Life Sci 851: 12-20.

7. Koeners MP, van Faassen EE, Wesseling S, de Sain-van der Velden M, Koomans HA, et al. (2007) Maternal supplementation with citrulline increases renal nitric oxide in young spontaneously hypertensive rats and has long-term antihypertensive effects. Hypertension 50: 1077-1084.
